# Handgrip Strength Values Depend on Tumor Entity and Predict 180-Day Mortality in Malnourished Cancer Patients

**DOI:** 10.3390/nu14102173

**Published:** 2022-05-23

**Authors:** Pascal Tribolet, Nina Kaegi-Braun, Carla Gressies, Annic Baumgartner, Karl-Heinz Wagner, Zeno Stanga, Philipp Schuetz

**Affiliations:** 1 Department of Health Professions, Bern University of Applied Sciences, 3008 Bern, Switzerland; pascal.tribolet@bfh.ch; 2 Department of Nutritional Sciences and Research Platform Active Ageing, University of Vienna, 1090 Vienna, Austria; karl-heinz.wagner@univie.ac.at; 3 Division of General Internal and Emergency Medicine, Medical University Department, Kantonsspital Aarau, 5001 Aarau, Switzerland; nina.kaegi@ksa.ch (N.K.-B.); carla.gressies@ksa.ch (C.G.); annic.baumgartner@ksa.ch (A.B.); 4 Division of Diabetes, Endocrinology, Nutritional Medicine and Metabolism, Inselspital, Bern University Hospital, University of Bern, 4001 Bern, Switzerland; zeno.stanga@insel.ch; 5 Medical Faculty, University of Basel, 4056 Basel, Switzerland

**Keywords:** handgrip strength, malnutrition, cancer, nutritional support, clinical outcomes

## Abstract

Background: Cancer-related malnutrition is a prevalent condition associated with a loss of muscle mass and impaired functional status, leading to immunodeficiency, impaired quality of life and adverse clinical outcomes. Handgrip strength (HGS) is a practical measure to assess muscle strength in individual patients during clinical practice. However, HGS reference values refer to populations of healthy people, and population-specific values, such as those in the population of cancer patients, still need to be defined. Methods: Within a secondary analysis of a previous randomized controlled nutritional trial focusing on hospitalized cancer patients at risk for malnutrition, we investigated sex-specific HGS values stratified by age and tumor entity. Additionally, we examined the association between HGS and 180-day all-cause mortality. Results: We included data from 628 cancer patients, which were collected from eight hospitals in Switzerland. Depending on the age of patients, HGS varied among female patients from 7 kg to 26 kg and among male patients from 20.5 kg to 44 kg. An incremental decrease in handgrip strength by 10 kg resulted in a 50% increase in 180-day all-cause mortality (odds ratio 1.52 (95%CI 1.19 to 1.94), *p* = 0.001). Conclusion: Our data provide evidence of the prognostic implications of HGS measurement in cancer patients and validate the prognostic value of handgrip strength in regard to long-term mortality. In addition, our results provide expected HGS values in the population of hospitalized malnourished cancer patients, which may allow better interpretation of values in individual patients.

## 1. Introduction

Malnutrition is a highly prevalent condition among oncology patients [1]. Up to 70% of cancer patients are at increased risk for malnutrition [2,3], a condition that is strongly associated with higher mortality and morbidity, functional decline, prolonged hospital stays and increased health care costs [4,5,6,7,8,9]. The pathophysiology of malnutrition in cancer patients is complex and involves different direct and indirect mechanisms, including inflammation, direct tumor effects, chemotherapy-induced effects and a decrease in appetite [10,11,12]. In addition, a reduced nutrient intake leads to protein and energy deficits, which in turn lead to muscle wasting and impairment of muscle strength [13,14].

Handgrip strength (HGS) measured through dynamometry is an important tool for the assessment of sarcopenia [15]. HGS has been proposed as an easy-to-use, noninvasive, objective and inexpensive tool to detect and monitor changes in nutritional status, and to predict functional decline during hospitalization and post-discharge [16,17,18,19]. HGS correlates with nutritional status and may detect changes in functional capacity in an early stage, before changes in the body composition are manifest [20]. Therefore, the use of HGS has been advocated by different international guidelines as an important adjunct in the assessment of malnutrition [21,22,23,24]. Moreover, for the population of cancer patients, studies have suggested that lower HGS is associated with higher risks for mortality and sarcopenia, as well as a decrease in quality of life (Qol) [25].

Based on published data of cancer patients, HGS reference values for this population are expected to be lower compared to healthy people [26,27,28,29,30], but there is a lack of data on reference values for this specific patient population. Herein, we investigated sex-specific HGS levels by tumor entity and additionally studied the prognostic information regarding 180-day all-cause mortality and other adverse outcomes from cancer patients included in the Effect of Early Nutritional Support on Frailty, Functional Outcome, and Recovery of Malnourished Medical Inpatients Trial (EFFORT) [31]. Knowledge of such data may provide health care workers with information about expected results when managing cancer patients in their clinical routine.

## 2. Material and Methods

### 2.1. Study Design and Setting

This is a secondary analysis of the EFFORT trial, which was a pragmatic, investigator-initiated, open-label, randomized controlled trial conducted in eight Swiss hospitals between April 2014 and February 2018. The original study investigated the effects of a protocol-guided individualized nutritional treatment algorithm on medical outcomes in patients at nutritional risk. The protocol and the main results [31,32], as well as several predefined secondary analyses [33,34,35,36,37,38,39,40,41,42], have been previously published. The EFFORT trial was registered with ClinicalTrials.gov, number NCT02517476.

The Ethics Committee of Northwest/Central Switzerland (EKNZ) approved the study protocol in January 2014 (EKNZ; 2014_001). The eight participating sites were secondary and tertiary care hospitals in Switzerland and included the University Clinic in Aarau, the University Hospital in Bern, the cantonal hospitals in Solothurn, Lucerne, St. Gallen, Baselland, Muensterlingen and the hospital in Lachen.

### 2.2. Patient Population

The methods of the trial have been previously published in detail [31,32]. In brief, for the current analysis, all patients with a cancer diagnosis (wither as the main diagnosis or a comorbidity, or both) from the original trial with available HGS measurements at the time of hospital admission were eligible. EFFORT enrolled consecutive adult patients with a Nutritional Risk Screening 2002 (NRS 2002) [43] total score ≥ 3 points, an expected length of hospital stay (LOS) ≥ 5 days and a willingness to provide informed consent. Patients initially admitted to a surgical unit or intensive care unit were excluded. Other exclusion criteria related to some diseases, including anorexia nervosa, acute pancreatitis, acute liver failure or cystic fibrosis, terminal condition, stem cell transplantation, history of gastric bypass surgery, contraindications for nutritional support, nutritional support at the time of admission and previous inclusion in the trial.

### 2.3. Assessment and Classification of Handgrip Strength

Grip strength data were collected at the time of admission by trained dieticians with a dynamometer (North Coast Medical Exacta™ Hydraulic Hand Dynamometer, North Coast Medical, Inc., 780 Jarvis Drive, Suite 100, Morgan Hill, CA 95037, USA [44]). The unit of measurement was kg, and measurements were performed in a seated position at the edge of the bed using the dominant hand at a 90° angle position without contacting any surface [45]. The patients performed three attempts, interrupted by a one-minute break, and the highest result was collected.

### 2.4. Outcomes

For this analysis, the primary endpoint was defined as 180-day all-cause mortality. We prespecified additional short-term and long-term secondary endpoints, including adverse clinical outcomes within 30 days (composite endpoint of the original trial including all-cause mortality, admission to intensive care unit (ICU), 30-day readmission rate, functional decline, length of hospital stay, non-elective hospital readmission and major complications (including nosocomial infection, respiratory failure, a major cardiovascular event and acute renal failure or gastrointestinal failure during hospitalization)) and activities of daily living assessed by Barthel Index. Further long-term secondary outcomes included QoL and incidence of falls during the 180-days follow-up period. QoL was assessed using: (a) the EuroQol Group 5-Dimension Self-Report Questionnaire (EQ-5D), which ranges from 0 to 1, with higher scores indicating better life quality, and (b) the EQ-5D visual analogue scale (VAS) from 0 to 100, with higher scores indicating better health status.

### 2.5. Statistical Analysis

Categorical variables are expressed as counts and percentages, and continuous variables as means and standard deviations. We performed descriptive statistics by calculating mean HGS according to tumor entities (hematological tumors, lung cancer, gastrointestinal tumors, prostate carcinoma, breast carcinoma and others (gynecological cancers, kidney and urothelial cancers, ear, nose and throat carcinoma, genital cancer, skin cancer, pleural mesothelioma and cancer of unknown primary and similar)) and age (10-year intervals) stratified by sex.

The association between sex-specific HGS and clinical outcome was investigated using logistic regression analyses for categorical variables with reporting of odd ratios (ORs) and linear regression for continuous variables with reporting of coefficients (Coef) and 95% confidence intervals (CI). We adjusted the results for important confounders (sex, age, weight, height, NRS 2002 score, center), several main diagnoses (cardiovascular, infectious, renal, frailty), various comorbidities (hypertension, chronic kidney disease, chronic heart failure, diabetes mellitus) and for randomization group.

All statistical analyses were performed with STATA 15.1 (Stata Corp, College Station, TX, USA). A *p* value < 0.05 (for a 2-sided test) was considered to indicate statistical significance.

## 3. Results

### 3.1. Patient Cohort

Of the initial population of 2028 patients included in the original trial recruiting patients from April 2014 to February 2018, we had complete data for 628 (368 male and 260 female) cancer patients from eight hospitals in Switzerland. The baseline characteristics for all patients included in this analysis, stratified according to sex, are shown in Table 1. Patients had a mean age of 72 years (±12.5), and 41.4% were females. The mean (SD) BMI was 24.6 (±4.8) with a similar distribution in both sexes, and the most common admission diagnosis was cancer (50.8%), followed by infection (21.0%). Patients had a high burden of comorbidities, including hypertension (49.8%), chronic renal disease (30.3%), coronary heart disease (24.8%), diabetes mellitus (19.9%) and chronic heart failure (11.6%). The most frequent types of cancer were hematological tumors (19.7%), lung cancer (16.4%), and gastrointestinal tumors (12.4%).

### 3.2. Handgrip Measurement in the Study Population

The overall mean (SD) HGS was 23.6 (±10.7 kg) with lower values in females (17.3 ± 6.3) compared to males (28.0 ± 10.8). Age, tumor entity and sex-specific HGS data are presented in Table 2. With higher age, the mean (SD) HGS decreased. In younger male cancer patients (<50 years), the mean (SD) HGS was 45.1 kg (±12.7 kg), while in patients ≥ 90 years, there was a mean (SD) HGS of 19.5 kg (±8.0 kg). For female cancer patients, the mean (SD) HGS values were lower, ranging from 23.1 kg (±8.9 kg) in young patients to 8.8 kg (±4.8 kg) in the oldest age group (≥90 years). Stratified by tumor entity, lung cancer patients had the highest mean HGS with 27.4 kg (±10 kg), which was consistent in both sexes (male: mean HGS of 30.8 kg (±9.6 kg), female: mean HGS of 18.9 kg (±4.5 kg)). In the female population, the lowest mean HGS was found in gastrointestinal tumor patients: 16.4 kg (±6.0 kg), whereas male patients had the lowest HGS with prostate carcinoma: 23.6 (±7.4 kg).

### 3.3. Association of Handgrip with Adverse Outcomes

In a second step, we investigated the prognostic value of HGS in this population of cancer patients stratified by sex (Table 3). In our overall adjusted statistical model, a 10 kg decrease in HGS was associated with a 50% increase in the risk of 180-day all-cause mortality (adjusted OR 1.52 (95% CI 1.19 to 1.94), *p* = 0.001). The effect was similar among male and female patients, but the association was only significant in male patients (adjusted OR 1.59 (95% CI 1.19 to 2.12), *p* = 0.002 vs. adjusted OR 1.54 (95% CI 0.89 to 2.65), *p* = 0.122 for female patients).

We also found significant associations between HGS and other clinical endpoints, namely 30-day all-cause mortality (adjusted OR 1.59 (95% CI 1.13 to 2.22), *p* = 0.007), admission to ICU (adjusted OR 2.58 (95% CI 1.08 to 6.16), *p* = 0.033), major complications (adjusted OR 1.65 (95% CI 1.09 to 2.51), *p* = 0.018), mean Barthel Index score (points) (adjusted Coef −1.44 (95% CI-2.56 to −0.33), *p* = 0.011) and incidence of one or more falls within 180 days (adjusted OR 1.58 (1.02 to 2.46), *p* = 0.04).

## 4. Discussion

This secondary analysis of a large, randomized trial found HGS to be highly predictive of long- and short-term mortality and other adverse outcomes among cancer patients. In line with previous research, HGS values depended on sex and patient age [26,27,36], but we additionally found important differences among different types of cancers. Our data provide important HGS reference values for the specific population of cancer patients, which may help future counseling of patients and interpretation of HGS results.

Through the additional stratification by tumor entity, a more precise assessment of functional status and muscle strength via HGS measurements is possible within the population of malnourished cancer patients. It may help to better understand the value of a single HGS measurement in an individual cancer patient and puts this measurement in the perspective of what is expected of the specific population. For this reason, our HGS data from a large, randomized controlled trial may contribute to a better classification of the functional status of malnourished cancer patients by HGS values. Nevertheless, our analysis is still limited by sample size within the different tumor entities, and larger studies would be useful to provide better estimates. Further investigations should also focus on the predictive value of HGS in different tumor entities stratified by age and sex.

Our analysis also showed a significant association of HGS in cancer patients and different clinical outcomes, such as all-cause mortality within 180 days, which is consistent with findings from our research group including other patient populations [36]. In fact, an incremental decrease in HGS by 10 kg resulted in more than doubling the risk for 180-d all-cause mortality among all tumor entities and sexes. These results persisted after adjustment for important cofounders, such as randomization, age, weight, height, NRS 2002, main diagnosis and comorbidities. These findings underline the prognostic value of HGS in malnourished cancer patients and are consistent with results from studies that also included patients with different diseases [36,41,46,47]. Additionally, our results show that, in the overall population, there is a significant association between the Barthel Index score and a decrease in HGS. As both are instruments for assessing functional status, this is an expected result, which was not stable in sex-specific subgroups. A decrease in HGS by 10 kg was also associated with other short-term endpoints, as shown in Table 3.

While the role of nutrition in cancer patients has received little attention, several studies observed a strong increase in mortality in patients with higher nutritional risk [41,48,49,50]. Indeed, patients with an NRS of ≥5 points had a 19% higher risk of long-term mortality compared to those with 3 points in a previous analysis [41]. Our data now suggest that, in addition to clinical information about weight and low appetite included in the NRS score, HGS is an additional parameter that helps providers understand the risk of a patient and may also help with decisions regarding the start of nutritional interventions. Importantly, we recently found that HGS was also predictive for treatment response, with patients in the lowest HGS ranges showing the best response rates [36].

The present analysis has several strengths worth mentioning, including the rather large population of patients with different types of cancer and the prospective gathering of data as part of the EFFORT trial [31,32]. High adherence to the study protocol in the main trial further increases the value of data collected. Limitations include the secondary analysis with limited power and the exploratory nature of our analyses, with the risk for model overfitting and type I error. Validation of our results is thus necessary. Further, we did not include critically ill and surgical cancer patients, which makes our findings only applicable to medical cancer patients. Since we had only limited information about the CKD stages, we did not consider a further stratification.

## 5. Conclusions

Our data provide evidence about the prognostic implications of HGS measurement in cancer patients and validate the prognostic value of HGS in regard to long-term mortality. In addition, our results provide expected HGS values in the population of hospitalized malnourished cancer patients, which may allow better interpretation of values in individual patients.

## Figures and Tables

**Table 1:** Baseline characteristics of malnourished cancer patients.

	Overall	Female	Male
*n*	628	260	368
Sociodemographic			
Age (years), mean (SD)	72.0 (12.5)	72.3 (11.5)	71.9 (13.2)
Nutritional status			
BMI (kg/m^2^), mean (SD)	24.6 (4.8)	24.6 (5.4)	24.6 (4.3)
Weight (kg), mean (SD)	70.7 (14.9)	65.3 (14.0)	74.4 (14.5)
Height (cm), mean (SD)	168.6 (8.8)	162.2 (6.5)	173.1 (7.2)
NRS 3	175 (27.9%)	72 (27.7%)	103 (28.0%)
NRS 4	200 (31.8%)	85 (32.7%)	115 (31.3%)
NRS 5	253 (40.3%)	103 (39.6%)	150 (40.8%)
Main diagnosis			
Cancer	319 (50.8%)	141 (54.2%)	178 (48.4%)
Infection	132 (21.0%)	44 (16.9%)	88 (23.9%)
Cardiovascular	34 (5.4%)	16 (6.2%)	18 (4.9%)
Frailty	45 (7.2%)	21 (8.1%)	24 (6.5%)
Lung	22 (3.5%)	7 (2.7%)	15 (4.1%)
Gastrointestinal	29 (4.6%)	15 (5.8%)	14 (3.8%)
Neurological/psychiatric	13 (2.1%)	5 (1.9%)	8 (2.2%)
Renal	11 (1.8%)	3 (1.2%)	8 (2.2%)
Metabolic	6 (1.0%)	3 (1.2%)	3 (0.8%)
Other	12 (1.9%)	4 (1.5%)	8 (2.2%)
Comorbidities			
Tumor	580 (92.4%)	237 (91.2%)	343 (93.2%)
Hypertension	313 (49.8%)	137 (52.7%)	176 (47.8%)
Chronic kidney disease (without kidney replacement therapy)	190 (30.3%)	71 (27.3%)	119 (32.3%)
Coronary heart disease	156 (24.8%)	47 (18.1%)	109 (29.6%)
Diabetes mellitus	125 (19.9%)	49 (18.8%)	76 (20.7%)
Chronic heart failure	73 (11.6%)	23 (8.8%)	50 (13.6%)
Chronic obstructive pneumopathypulmonary disease	70 (11.1%)	24 (9.2%)	46 (12.5%)
Peripheral arterial vascular disease	43 (6.8%)	13 (5.0%)	30 (8.2%)
Stroke	39 (6.2%)	10 (3.8%)	29 (7.9%)
Dementia	14 (2.2%)	6 (2.3%)	8 (2.2%)
Tumor entity			
Hematological tumors	124 (19.7%)	53 (20.4%)	71 (19.3%)
Lung cancer	103 (16.4%)	29 (11.2%)	74 (20.1%)
Gastrointestinal tumors	78 (12.4%)	30 (11.5%)	48 (13.0%)
Prostate carcinoma	62 (9.9%)		62 (16.8%)
Breast carcinoma	56 (8.9%)	55 (21.2%)	1 (0.3%)
Other *	205 (32.6%)	93 (35.8%)	112 (30.4%)
Handgrip strength (kg), mean (SD)			
Overall HGS	23.6 (10.7)	17.3 (6.3)	28.0 (10.8)

* Gynecological cancers, kidney and urothelial cancers, ear, nose and throat carcinoma, genital cancer, skin cancer, pleural mesothelioma and cancer of unknown primary.

**Table 2:** Handgrip strength according to tumor entity and age.

	Overall (*n* = 628)			Female (*n* = 260)			Male (*n* = 368)		
Age (year)	*n*	HGS Mean (kg) (SD)	*p*	*n*	HGS Mean (kg) (SD)	*p*	*n*	HGS Mean (kg) (SD)	*p*
<50	30	38.5 (15.4)	<0.001	21	23.1 (8.9)	<0.001	9	45.1 (12.7)	<0.001
50–59	66	29.6 (10.3)		40	23.2 (6.0)		26	33.7 (10.5)	
60–69	119	24.4 (9.9)		66	19.0 (6.6)		53	28.8 (10.0)	
70–79	233	23.3 (8.9)		135	17.3 (6.3)		98	27.6 (8.0)	
80–89	146	19.6 (9.2)		84	14.5 (5.3)		62	23.4 (9.6)	
≥90	34	15.7 (8.7)		22	8.8 (4.8)		12	19.5 (8.0)	
Tumor entity									
Hematological tumors	124	23.1 (11.0)	<0.001	53	18.3 (7.0)	0.48	71	26.7 (12.1)	0.002
Lung cancer	103	27.4 (10)		29	18.9 (4.5)		74	30.8 (9.6)	
Gastrointestinal tumors	78	24.1 (11.3)		30	16.4 (6.0)		48	28.9 (11.2)	
Prostate carcinoma	62	23.6 (7.4)		-	-		62	23.6 (7.4)	
Breast carcinoma	56	17 (17.4)		55	16.9 (7.4)		1	18	
Other *	205	23.7 (11.5)		93	16.9 (7.4)		112	29.3 (11.3)	

Abbreviations: HGS, handgrip strength; SD, standard deviation. * Gynecological cancers, kidney and urothelial cancers, ear, nose and throat carcinoma, genital cancer, skin cancer, pleural mesothelioma and cancer of unknown primary.

**Table 3:** Association of handgrip strength with short- and long-term outcomes stratified by sex.

	HGS Mean (SD), Patients with No Event	HGS Mean (SD), Patients with Event	HGS Decrease Cont (−10 kg)	HGS Decrease Cont (−10 kg)
All patients			Unadjusted OR or Coef (95% CI), *p*-value	* Adjusted OR or Coef (95% CI), *p*-value
Primary endpoint				
180-day all-cause mortality	24.42 (11.13)	22.35 (9.99)	1.2 (1.03 to 1.41) *p* = 0.019	1.52 (1.19 to 1.94), *p* = 0.001
Short-term endpoints (30 days)				
All-cause mortality	23.81 (10.65)	22.15 (11.37)	1.16 (0.92 to 1.48) *p* = 0.211	1.59 (1.13 to 2.22), *p* = 0.007
Adverse outcome	23.82 (10.73)	23.17 (10.76)	1.06 (0.9 to 1.24) *p* = 0.481	1.23 (0.98 to 1.54), *p* = 0.077
Admission to the intensive care unit	23.71 (10.8)	19 (6.1)	1.64 (0.89 to 3.01) *p* = 0.114	2.58 (1.08 to 6.16), *p* = 0.033
Non-elective hospital readmission	23.42 (10.71)	25.1 (10.92)	0.87 (0.7 to 1.08) *p* = 0.211	0.84 (0.61 to 1.15), *p* = 0.283
Any major complication	23.87 (10.85)	20.53 (8.84)	1.39 (1.02 to 1.89) *p* = 0.038	1.65 (1.09 to 2.51), *p* = 0.018
Decline in functional status of ≥10% *	23.75 (10.5)	22.93 (11.9)	1.08 (0.88 to 1.31) *p* = 0.475	1.18 (0.89 to 1.58), *p* = 0.254
Mean length of stay (days)	-	-	0.22 (−0.29 to 0.73) *p* = 0.398	0.65 (−0.08 to 1.37), *p* = 0.081
Mean Barthel Index score (points)	-	-	−1.69 (−2.48 to −0.9) *p* < 0.001	−1.44 (−2.56 to −0.33), *p* = 0.011
Long-term endpoints (180 days)				
Mean EQ-5D VAS (points)	-	-	−0.81 (−2.87 to 1.25) *p* = 0.442	−1.2 (−4.14 to 1.75), *p* = 0.425
Mean EQ-5D index (points)	-	-	−0.02 (−0.03 to 0) *p* = 0.027	−0.01 (−0.03 to 0.01), *p* = 0.363
Incidence of one or more falls	23.84 (10.69)	20.37 (10.53)	1.41 (1.04 to 1.91) *p* = 0.027	1.58 (1.02 to 2.46), *p* = 0.04
Female patients				
Primary endpoint				
180-day all-cause mortality	18.14 (7.08)	15.9 (6.31)	1.62 (1.11 to 2.37) *p* = 0.013	1.54 (0.89 to 2.65), *p* = 0.122
Short-term endpoints (30 days)				
All-cause mortality	17.68 (6.79)	14.33 (7.17)	2.05 (1.12 to 3.74) *p* = 0.02	2.26 (1.03 to 4.95), *p* = 0.041
Adverse outcome	17.39 (6.77)	17.24 (7.22)	1.03 (0.7 to 1.52) *p* = 0.876	1.31 (0.8 to 2.15), *p* = 0.275
Admission to the intensive care unit	17.32 (6.94)	18.43 (4.83)	0.79 (0.26 to 2.37) *p* = 0.673	1.33 (0.3 to 5.83), *p* = 0.704
Non-elective hospital readmission	17.05 (6.89)	19.63 (6.55)	0.57 (0.32 to 1.01) *p* = 0.055	0.75 (0.37 to 1.55), *p* = 0.444
Any major complication	17.4 (6.84)	16.63 (7.59)	1.18 (0.6 to 2.32) *p* = 0.638	1.55 (0.67 to 3.57), *p* = 0.304
Decline in functional status of ≥10%	17.66 (6.84)	15.51 (6.98)	1.58 (0.95 to 2.62) *p* = 0.076	1.23 (0.64 to 2.39), *p* = 0.532
Mean length of stay (days)	-	-	0.33 (−0.88 to 1.53) *p* = 0.596	0.43 (−1.06 to 1.92), *p* = 0.569
Mean Barthel Index score (points)	-	-	−2.89 (−4.92 to −0.86) *p* = 0.005	−2.44 (−4.94 to 0.06), *p* = 0.056
Long-term endpoints (180 days)				
Mean EQ-5D VAS (points)	-	-	−2.91 (−7.42 to 1.6) *p* = 0.204	−1.47 (−6.89 to 3.95), *p* = 0.592
Mean EQ-5D index (points)	-	-	−0.05 (−0.09 to −0.01) *p* = 0.013	−0.04 (−0.09 to 0.01), *p* = 0.084
Incidence of one or more falls	17.59 (6.84)	13.56 (6.13)	2.38 (1.14 to 4.95) *p* = 0.021	3.57 (1.36 to 9.41), *p* = 0.01
Male patients				
Primary endpoint				
180-day all-cause mortality	29.33 (11.26)	26.23 (9.79)	1.32 (0.01 to 1.63) *p* = 2.69	1.59 (1.19 to 2.12), *p* = 0.002
Short-term endpoints (30 days)				
All-cause mortality	28.29 (10.73)	26.38 (11.01)	1.19 (0.25 to 1.61) *p* = 1.14	1.61 (1.09 to 2.38), *p* = 0.016
Adverse outcome	28.54 (10.66)	27 (10.96)	1.15 (0.2 to 1.41) *p* = 1.28	1.18 (0.91 to 1.55), *p* = 0.218
Admission to the intensive care unit	28.18 (10.77)	19.67 (7.76)	2.77 (0.05 to 7.73) *p* = 1.95	4.28 (0.83 to 22.16), *p* = 0.083
Non-elective hospital readmission	27.92 (10.65)	29.02 (11.78)	0.91 (0.53 to 1.22) *p* = −0.62	0.79 (0.54 to 1.15), *p* = 0.217
Any major complication	28.47 (10.83)	23.09 (8.77)	1.76 (0.01 to 2.71) *p* = 2.58	1.61 (0.95 to 2.73), *p* = 0.08
Decline in functional status of ≥10% *	28.24 (10.47)	27.14 (12.09)	1.1 (0.45 to 1.42) *p* = 0.76	1.19 (0.86 to 1.66), *p* = 0.297
Mean length of stay (days)	-	-	0.47 (0.17 to 1.15) *p* = 1.38	0.59 (−0.28 to 1.46), *p* = 0.182
Mean Barthel Index score (points)	-	-	−1.67 (0 to −0.69) *p* = −3.35	−0.96 (−2.2 to 0.29), *p* = 0.132
Long-term endpoints (180 days)				
Mean EQ-5D VAS (points)	-	-	−1.27 (0.39 to 1.63) *p* = −0.86	−0.45 (−4.18 to 3.28), *p* = 0.813
Mean EQ-5D index (points)	-	-	−0.01 (0.47 to 0.01) *p* = −0.73	0 (−0.03 to 0.02), *p* = 0.810
Incidence of one or more falls	28.42 (10.69)	23.78 (10.67)	1.61 (0.02 to 2.37) *p* = 2.4	1.29 (0.78 to 2.11), *p* = 0.32

Abbreviations: OR, odds ratio; Coef, coefficient; SD, standard deviation; EQ-5D, EuroQol Group 5-Dimension Self-Report Questionnaire; HGS, handgrip strength; VAS, visual analogue scale. * Adjusted for randomization, age, weight, height, NRS 2002, center, main diagnosis (cardiovascular, infection, renal disease, failure to thrive) and comorbidities (hypertension, chronic kidney failure, chronic heart failure, diabetes mellitus) and for randomization group.

## Data Availability

The datasets used and analyzed during the current study are available from the corresponding author on reasonable request.
